# Benchmarking 3D electron diffraction strategies for ceramics

**DOI:** 10.1107/S2052252526003155

**Published:** 2026-04-27

**Authors:** Yann Schmitt, Sergi Plana-Ruiz, Yaşar Krysiak

**Affiliations:** a Institute of Inorganic Chemistry Leibniz Universität Hannover Callinstraße 9 30167Hannover Germany; b Unitat de Cristal·lografia i Mineralogia, Department de Geologia, Universitat Autònoma de Barcelona, 08193 Cerdanyola del Vallès, Catalonia, Spain; Istituto Italiano di Tecnologia, Italy

**Keywords:** ceramics, dynamical scattering, NASICON, almandine, Kikuchi lines, 3D ED, precession electron diffraction, continuous-rotation electron diffraction

## Abstract

Three-dimensional electron diffraction (3D ED) has become increasingly popular over the last two decades, challenging the limits of established single-crystal X-ray diffraction experiments. In particular, continuous-rotation and precession-assisted protocols have been established as important tools in the collection of electron diffraction data, and the data for most of the crystal structures solved by 3D ED nowadays are acquired with one of these two methods. This is particularly true for ceramic materials, where 3D ED allows deep insights into complex structure–property relationships. As ceramic syntheses tend to yield thick and highly crystalline particles, one issue in the refinement against electron diffraction data is the effect of coherent inelastic scattering. While dynamical refinement procedures allow the simulation of multiple scattering events, in general they ignore the inelastic contribution. This work aims to evaluate the impact of dynamical inelastic effects on the diffraction data of ceramics and how their overall quality depends on the measurement strategy used. This was done by comparing the structure models derived from the same crystals under similar experimental conditions of three ceramic compounds, namely NATP [Na_1+*x*_Al_*x*_Ti_2−*x*_(PO_4_)_3_], LATP [Li_2−*x*_Al_*x*_Ti_2−*x*_(PO_3_)_4_] and (Fe_3_Al_2_[SiO_4_]_3_), based on precession, continuous-rotation and stepwise static tilt data. The different methods allowed the detection of low-occupancy sites in the difference electrostatic potential maps corresponding to mobile sodium and lithium ions, paving the way for future studies on their migration behaviour in solid-state electrolytes.

## Abstract 2

Three ceramic samples were analysed using three-dimensional electron diffraction and a comparison of the different data collection strategies revealed that coherent inelastic scattering effects hamper the interpretation of difference electrostatic potential maps and can have a significant impact on the refinement statistics.

## 1. Introduction

Structural investigations on ceramic materials by electron microscopy have a long history, combining high-resolution imaging with probing the internal microstructure by selected area electron diffraction (SAED) and the chemical composition by energy dispersive X-ray (EDX) (Falk, 1998 ▸) and electron energy loss spectroscopy (EELS) (Keast, 2012 ▸). About two decades ago, Kolb *et al.* suggested measuring arbitrarily oriented crystals by automated tilt-series experiments, recording off-zone reflections and thus allowing a 3D reconstruction of the diffraction space (Kolb *et al.*, 2007 ▸; Kolb *et al.*, 2008 ▸). Since then, other research groups have suggested same-principle-based approaches with different experimental setups, working out the specific implementation details and accounting for the many subtleties in processing electron diffraction data. These new methods are collectively referred to as three-dimensional electron diffraction (3D ED) (Gemmi *et al.*, 2019 ▸), and among them precession electron diffraction (PED) and continuous-rotation electron diffraction have emerged as the most frequently used data acquisition techniques for structure determination, as shown in a recent literature survey (Klar *et al.*, 2023 ▸). Continuous-rotation 3D ED, also referred to as cRED (Nederlof *et al.*, 2013 ▸), MicroED (Nannenga *et al.*, 2014 ▸) or IEDT (Gemmi *et al.*, 2015 ▸), is best understood as equivalent to rotational scanning from the viewpoint of X-ray crystallographers, and the growing accessibility of faster and more sensitive electron detectors from 2010 to 2020 further sparked the method’s development. On the other hand, inspired by the Buerger precession technique and first demonstrated in the context of electron diffraction by Vincent & Midgley (1994 ▸), the idea of conical beam rocking combined with sequentially tilting the crystal became popular as precession-assisted electron diffraction tomography (Mugnaioli *et al.*, 2009 ▸). Owing to their high beam stability, it comes as no surprise that ceramics were among the first materials to be studied by 3D ED (Rozhdestvenskaya *et al.*, 2010 ▸; Mugnaioli *et al.*, 2012 ▸; Andrusenko *et al.*, 2015 ▸), and the method rapidly grew into a now well established tool for the analysis of even complex and strongly disordered ceramic and mineral structures (Zhao *et al.*, 2017 ▸; Gollé-Leidreiter *et al.*, 2024 ▸; Plana-Ruiz *et al.*, 2024 ▸).

The biggest advantage of the 3D ED methodology probably lies in the accessible sample volume, enabling the structure determination of particles on the nanoscale and bypassing the problem of growing micron-sized single crystals of sufficient quality. One such example is provided by the ion-conducting ceramics from the NASICON family (Goodenough *et al.*, 1976 ▸), where single-crystal structure analysis is essential for the study of the structural features that enable the high ion-conducting properties of the framework. These include interstitial crystallographic sites occupied as a result of Na^+^ and Al^3+^ doping and not observable in laboratory powder X-ray diffraction (XRD). Crystals sufficient in size for single-crystal X-ray diffraction (SC-XRD) have to be grown laboriously over several weeks using long-term sintering techniques (Redhammer *et al.*, 2016 ▸). In theory, 3D ED allows the analysis of such subtle structural features from small single crystals (Palatinus *et al.*, 2017 ▸).

Owing to the large scattering cross section of electrons, however, the quality of the structure model that is obtained is often limited by strong dynamical scattering, leading to non-linear deviation of the reflection intensities and violation of the extinction rules (Henderson, 1995 ▸). For purely elastically scattered electrons, the refinement routine from arbitrary patterns collected by means of 3D ED taking dynamical scattering theory into account, termed dynamical refinement, allows one to obtain figures of merit like those from SC-XRD experiments (Palatinus *et al.*, 2019 ▸). In this context, experimental reflection intensities are fitted in a frame-based treatment against simulated dynamical intensities calculated from a Bloch-wave approach. Initially developed for data collected with the aid of precession, datasets measured in a continuous or stepwise fashion now allow for dynamical refinement as well, employing the concept of overlapping virtual frames (OVFs) (Klar *et al.*, 2023 ▸).

While these developments mark important steps in approaching SC-XRD quality, specific scattering events are not yet routinely considered in the refinement strategies that are employed. The simulation of inelastic scattering is normally enabled by an imaginary absorption term in the structure factor and has been shown to be neglectable for ED (Mendis, 2024 ▸), whereas the impact of coherent inelastic scattering remains largely unaddressed. Such scattering can be schematically understood as inelastically scattered electrons fulfilling the Bragg condition in a second scattering event, giving rise to pairs of Kikuchi lines. Cleverley & Beanland (2023 ▸) noticed that a small fraction of their dynamically refined continuous-rotation data had multiple peaks in their rocking curves, while also observing Kikuchi lines disturbing these reflections in the diffraction patterns. Apart from altering Bragg reflections, it was shown that Kikuchi lines also pose problems in local-order analysis, where a strongly modulated background hinders the detection of diffuse scattering intensities in 3D difference pair distribution function (3D-ΔPDF) analysis (Schmidt *et al.*, 2023 ▸). Precautions in the data collection can be taken by preparing thin lamellas by focused ion beam (FIB) milling or energy filtering (Thomas *et al.*, 2024 ▸), yielding good signal-to-background patterns. Unfortunately, these two approaches are not always practicable and post diffuse background subtractions become progressively more challenging when confronted with a complex, anisotropic diffuse background in the form of Kikuchi lines.

In this work, we report the impact of different diffraction pattern recording strategies on 3D ED data collected from sub-micrometre ceramic particles. As representatives of ceramic and mineral materials, two NASICON-related phases and almandine (Novak & Gibbs, 1971 ▸) were chosen for 3D ED measurements. To separate intrinsic properties like crystallinity, mosaicity and thickness differing between crystals from the used data collection technique, successive measurement series were performed on the same crystal retaining similar illumination and tilting conditions. The measurements were performed at two different microscope setups employing different acceleration voltages and acquisition software for the data collection. These conditions paved the way for a deeper analysis of the retrieved structure models, difference electrostatic potential (DESP) maps calculated from the difference Fourier synthesis, and individual reflection statistics from different 3D ED methods.

## 2. Experimental

### 2.1. Synthesis of NASICON ceramics

Li_1.3_Al_0.3_Ti_1.7_(PO_3_)_4_ (LATP) was synthesized via a sol–gel route similar to the one described by Bucharsky *et al.* (2015 ▸). The chemicals (reagent quality) were added in stoichiometric quantities, while the Ti salt was used in small excess. The Al(III) and Li(I) precursors, Al(NO_3_)_3_·9H_2_O and Li(C_2_H_3_O)·H_2_O, were dissolved in water under constant stirring. Ti(IV) isopropoxide (Ti[OCH(CH_3_)_2_]_4_) was added dropwise to form a loose TiO_2_ network. An aqueous NH_4_H_2_(PO_4_)_3_ solution was added dropwise under constant stirring. The gel was aged for 48 h at room temperature before calcinating the amorphous powder at 400°C and 800°C for 8 h at each temperature. The powder was densified via spark plasma sintering (SPS) at 1100°C, resulting in a dense piece of the ceramic. Small amounts of powder were scraped off the surface, containing both the orthorhombic Li_1+*x*_Al_*x*_Ti_2−*x*_(PO_3_)_4_ and Na_1.3_Al_0.3_Ti(PO_4_)_3_ (NATP) phases.

### 2.2. Origin of the mineral almandine

The mineral almandine Fe_3_Al_2_[SiO_4_]_3_ originates from Australia. A hydraulic press was used to break off a small piece of the mineral, which was then ground in an agate mortar. The powder and pestle were cooled with liquid nitro­gen to increase the brittleness of the sample. Elemental analysis via energy dispersive X-ray (EDX) spectroscopy confirmed the chemical composition of the garnet mineral. Larger particles additionally contained both Mn and Mg in minor proportions, suggesting the presence of other pyralspite garnet types (see Fig. S1).

### 2.3. Scanning electron microscopy

Elemental analysis of the almandine sample was carried out in a Hitachi Regulus SU8200 scanning electron microscope equipped with an Oxford Ultim Max 100 EDX detector. The microscope was operated at an acceleration voltage of 20 kV to excite all possible energy peaks. A scintillator-type Everhart–Thornley secondary electron detector was used to record images, serving as reference for point and area scans collected at a take-off angle of 45°.

### 2.4. Transmission electron microscopy

The measurements were carried out at two different electron microscope setups utilizing different data acquisition software. First, a Jeol F200 ColdFEG TEM operated at 200 kV was used. The diffraction patterns were acquired with a Gatan OneView camera (16 bit, 4096 × 4096 pixels) at different exposure times (0.25 s to 0.5 s). The detector’s read-out time was measured and amounted to 1 ± 1 ms when recording 100 frames. The powder sample was dispersed in ethanol and a single droplet was placed onto a carbon-coated Cu TEM grid. The grid was loaded on a Jeol analytical tomography holder and measurements were performed at room temperature. The data sets were acquired using the *Fast-ADT* (FADT) module developed for Jeol and FEI microscopes, currently available as a Digital Micrograph plug-in from GitHub (sergiPlana/TEMEDtools) (Plana-Ruiz *et al.*, 2020 ▸). After screening the grid for potential crystals, a tracking file for a promising particle was recorded in TEM mode. Subsequently, this file was used to automatically track the crystal while collecting the 3D ED dataset. The different measurements were carried out successively, attempting to retain similar experimental conditions. During all measurements, it was aimed to tilt over the largest angular range possible. A condenser aperture of 10 µm and mild illumination conditions were chosen to produce a 200 nm quasi-parallel beam following custom-made alignments (Plana-Ruiz *et al.*, 2018 ▸). Beam precession was achieved with the P2010 signal generator provided by NanoMEGAS SPRL. As a second experimental setup, a Hitachi 7800 microscope operated at 120 kV was used. The TEM grid was loaded on an HT7800-SS tilt holder and the *eHermelin* software was employed to collect 3D ED data following the FADT approach in TEM mode. Diffraction patterns were recorded on an Emsis XAROSA CMOS camera (14 bit, 5120 × 3840 pixels). A condenser aperture of 10 µm and a beam size of 400 nm were chosen to provide a quasi-parallel beam. Diffraction patterns were collected with different exposure times (0.5 s and 1 s) at room temperature. More details about the data acquisition are given in Tables S1, S2 and S3.

### 2.5. Software used in 3D ED data analysis

ED patterns in tif format were processed with *PETS2* (Palatinus *et al.*, 2019 ▸). The structures were solved *ab initio* using the charge flipping algorithm implemented in the program *Superflip* (Palatinus & Chapuis, 2007 ▸) or via direct methods in *SIR2014* (Burla *et al.*, 2015 ▸). Refinements using the kinematical approximation were performed in *JANA2020* (Petříček *et al.*, 2023 ▸). The dynamical refinement was performed using the Bloch-wave-based *Dyngo* module, also implemented in *JANA2020*. The root-mean-square deviation from reference atoms (RMSD) was calculated with *SIR2014*. Projections of the crystal structures were generated with *VESTA3* (Momma & Izumi, 2008 ▸).

## 3. Electron diffraction acquisition modules

**Figure 1:**
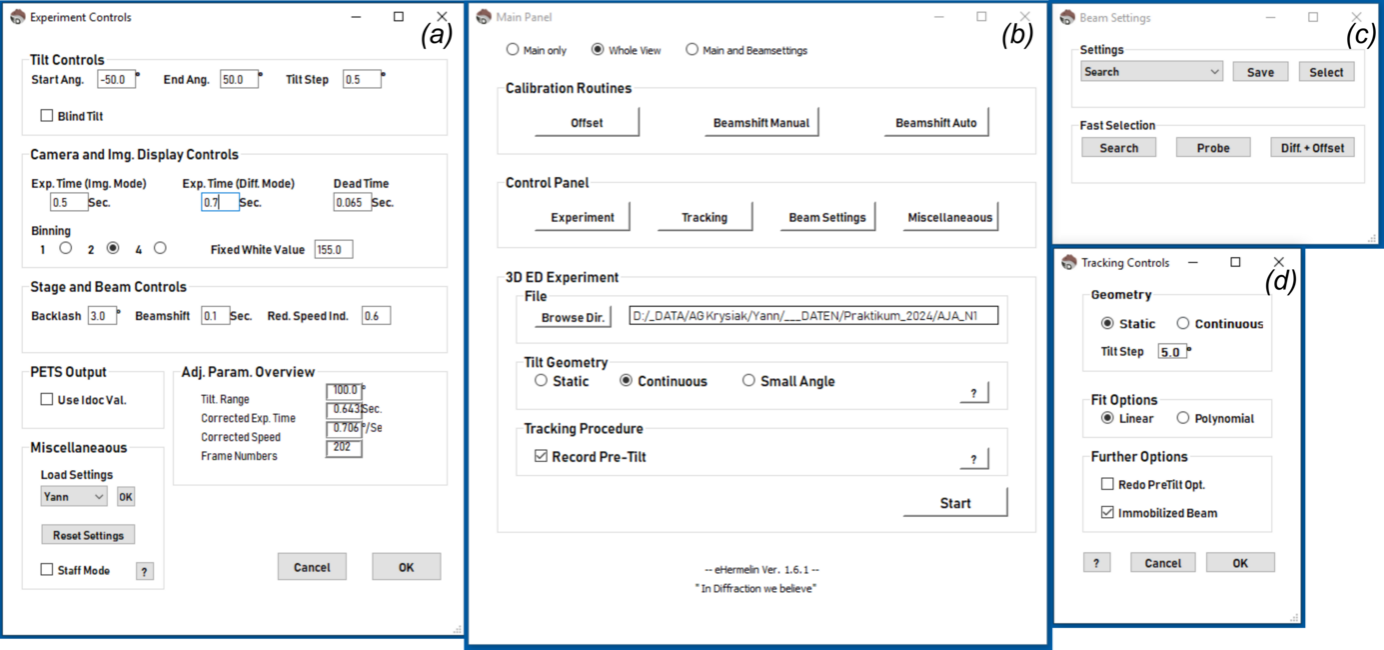
User interface of *eHermelin*. Different panels allow experimental control over (*a*) stage (angular tilt range, tilt step increment, step motor backlash) and camera parameters (exposure and dead times, binning and display options), (*b*) the data acquisition technique used (stepwise or continuous rotation), calibration routines and file management, (*c*) beam settings for the crystal search, tracking and diffraction mode and (*d*) crystal tracking procedure (scan type and extrapolation options).

Continuous recording relies strongly on stable goniometric stages as well as sample holders, yet even tomography holders specifically designed for high tilts are commonly affected by mechanical issues. This results in crystals moving out of the fixed area illuminated by the electron beam when tilting over large angular ranges, requiring tracking routines in order to keep the crystal illuminated for the whole tilt range. Here, the acquisition approach introduced by Gemmi *et al.* (2015 ▸) and further extended by Plana-Ruiz *et al.* (2020 ▸) was used. Briefly, this consists of a first tilt scan of the stage where images are collected to identify the dependence of the crystal position with respect to the tilt angle, and a second one where the 3D ED data are acquired by automatically shifting the beam to follow the crystal along the entire angular range. Two acquisition programs were used in this work for the continuous data collection: *Fast-ADT* (Plana-Ruiz *et al.*, 2020 ▸) and *eHermelin*. *Fast-ADT* is a Gatan Digital Micrograph plug-in readily available to work on Jeol and Thermo Fisher microscopes but limited to Gatan detectors. In this context, the Python-based software suite *eHermelin*, operating in principle on Thermo Fisher, Jeol and Hitachi microscopes in combination with a huge variety of CCD, CMOS and hybrid pixel electron detectors, is introduced in this work as a cross-platform solution for continuous-rotation data collection (Fig. 1 ▸). The *SerialEM* interface (Mastronarde, 2005 ▸), running as a background application and allowing wide microscope and camera control, makes the program code easily adaptable to other experimental setups. *eHermelin* allows for 3D ED experiments in continuous-rotation and stepwise tilt modes by using a single GUI (see Fig. 1 ▸). The program’s main output consists of an image stack containing the diffraction patterns and an input file for direct data processing in *PETS2*. A beam-shift alignment for converting deflector current changes into image shifts is implemented. The experimental procedure is facilitated by storable beam settings for the crystal search, the acquisition of tracking images and the final diffraction experiment. Along with a fast switch between the different modes, this ensures that the beam-alignment routine is up-to-date for a specific set of illumination conditions when reloading the settings during the experiment.

The *eHermelin* software further introduces utilities for issues that are often overlooked in 3D ED data collection: (i) a correction accounting for excitation changes in the projective lenses, most prominent when changing from imaging to diffraction mode, resulting in an image shift; (ii) a backlash correction accounting for sample shifts when changing the direction of rotation of the goniometric stage; (iii) a correction of the exposure time and selection of possible goniometric stage velocities provided by the microscope in respect to a user-given tilt-step increment; (iv) the live query of tilt angles corresponding to diffraction patterns in contrast to a hard input of post-calculated tilt angles; and (v) blind tilts prior to the experimental and tracking tilt scan, enhancing the reproducibility of the stage movement. The software has already been used successfully for the study of different materials, for example hybrid perovskites (Bahnmüller *et al.*, 2025 ▸; Dahlke *et al.*, 2025 ▸). Besides a stable goniometric stage and crystal tracking routine, the time resolution of the detector system should be consistently low, as long read-out times result in missing wedges in the reconstructed diffraction space of continuously acquired data (see Fig. S2). At the Hitachi HT7800 microscope, the provided camera plug-in (beta version) used for communication between the Emsis XAROSA camera and *SerialEM* software exhibited long internal communication delays, which in turn resulted in read-out times of more than 100 ms. These led to increased missing wedges when measuring at high stage velocities, so low angular tilt velocities were favoured. However, *eHermelin* considers the detector read-out time in the geometrical description of the recorded data.

## 4. Results

### 4.1. Data reduction

**Figure 2:**
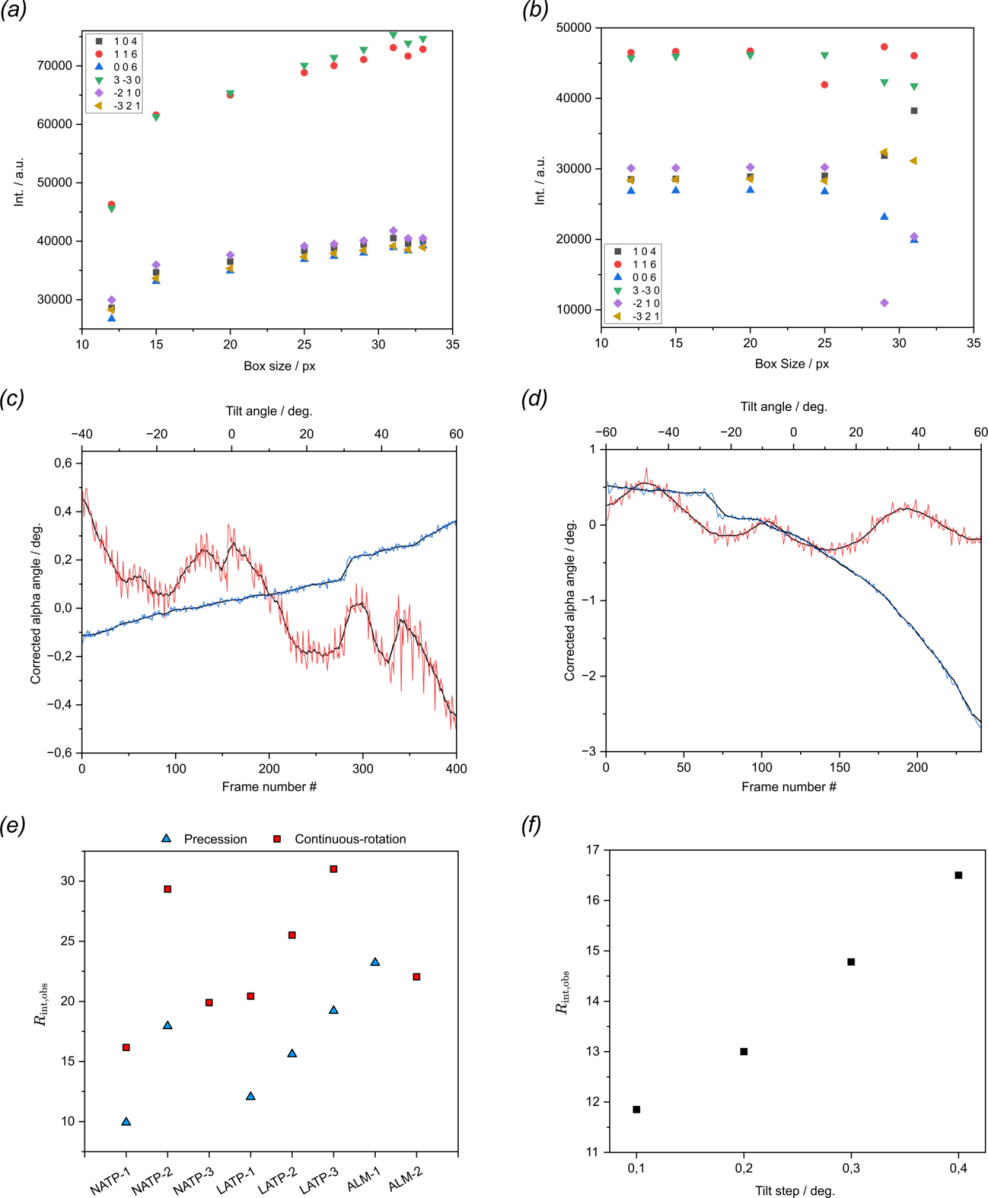
Collected intensity on single frames corresponding to strong reflections against user-defined box sizes integrated by means of the (*a*) sum counts and (*b*) fit profile method. (*c*) Frame-by-frame tilt correction plots of the alpha angle for LATP-3 measured with a Jeol analytical tomography holder and *(d*) ALM-2 measured with a Hitachi HT7800 single tilt holder. Data points in red correspond to angular corrections for tilt angles directly retrieved during acquisition, while data points in blue correspond to those calculated according to an initial tilt angle, tilt velocity and experiment time. The angle movement was smoothed out using a moving average of six frames (1.5°/3°). (*e*) Merged error *R*_int_ for different crystals measured either by precession or continuous-rotation ED. (*f*) Merged error *R*_int_ for different tilt increments in the stepwise static tilt experiment.

3D ED data from different ceramics (LATP/NATP and almandine) were collected from different crystals by continuous-rotation and precession ED. These were labelled as NATP-1, NATP-2, NATP-3, LATP-1, LATP-2, LATP-3, LATP-4, ALM-1 and ALM-2 (see Table 1 ▸ for an overview of the samples and the acquisition methods used). Whenever reasonable, similar parameters were used during the data reduction routine. This included an even mask size in the peak search procedure, same correlation ranges and weights for frame scaling and the same determination method in integrating the reflection spot intensities (see Section S1). We could show that using the standard peak-background method, which sums up the counts in a mask of pre-defined size and subtracts an interpolated background, fails to capture the complete intensity information of strong reflections [see Fig. 2 ▸(*a*)]. An integration box that is too small results in lost reflection intensity, while one that is too large leads to overlap of neighbouring reflections and undetected weak reflections. Thus, an average profile function was scaled to fit the individual reflection spots, a method shown to be mostly independent of the box size [see Fig. 2 ▸(*b*)]. To ensure an equal indexing of reflections for later investigations, the same crystallographic reference system was chosen for all data sets collected from the same crystal. During data reconstruction, care was taken to ensure that saturated reflection intensities were reliably identified despite software binning and were therefore falsely not extracted (see Fig. S3). Random and systematic deviations in the experimentally determined diffraction pattern orientations can be corrected retrospectively, and in this context some systematic features could be observed for the corrected orientations of the ED patterns in the case of all continuously acquired data sets. The tilt angle associated with a given diffraction pattern tends to be more error-prone in case of continuous-rotation protocols, where the angles are either calculated afterwards or retrieved by a query in the software after a certain elapsed tilt time for a given stage velocity, which is wrongly assumed to be constant.

While the latter leads to improved *R*_int_ factors compared with the calculated input, strong noise was observed for both microscopes in the tilt correction plots [see Fig. 2 ▸(*c*) and (*d*)]. The strong fluctuation was not observed for the other two orientation angles (see Fig. S4), therefore this is most likely not an artefact of an unstable goniometric stage. The noise could be accounted for by a delayed and slightly varying response time in the software query loop, therefore a smoothing function does not necessarily improve the reflection integration. All different data collection techniques ensured a successful indexing and determination of the unit-cell metrics for all measured data sets. The lattice parameters were similar for precession-assisted and continuously acquired data (Table S4) and consistent with reported literature data derived from XRD (Mouahid *et al.*, 2000 ▸; Kee *et al.*, 2011 ▸). The merging error *R*_int_ for the expected Laue group was on average 10% lower for data acquired by precession ED [see Fig. 2 ▸(*e*)]. Among the different crystals, NATP-1 was chosen for measurements in a stepwise static tilt fashion using tilt increments of 0.1°. The processed data yielded a reasonable low *R*_int_ value of 11.85% and allowed for successful determination of the unit-cell parameters. By only considering every second (0.2°), third (0.3°) and fourth (0.4°) frame, the maximum theoretical tilt increment for successful indexing was sought. While the number of indexed reflection peaks declined with increasing tilt increment, the data were sufficient for unit-cell determination even at larger tilt steps. Consequently, the merged error *R*_int_ converged to higher values [see Fig. 2 ▸(*f*)].

### 4.2. Structure determination of the ceramics

**Figure 3:**
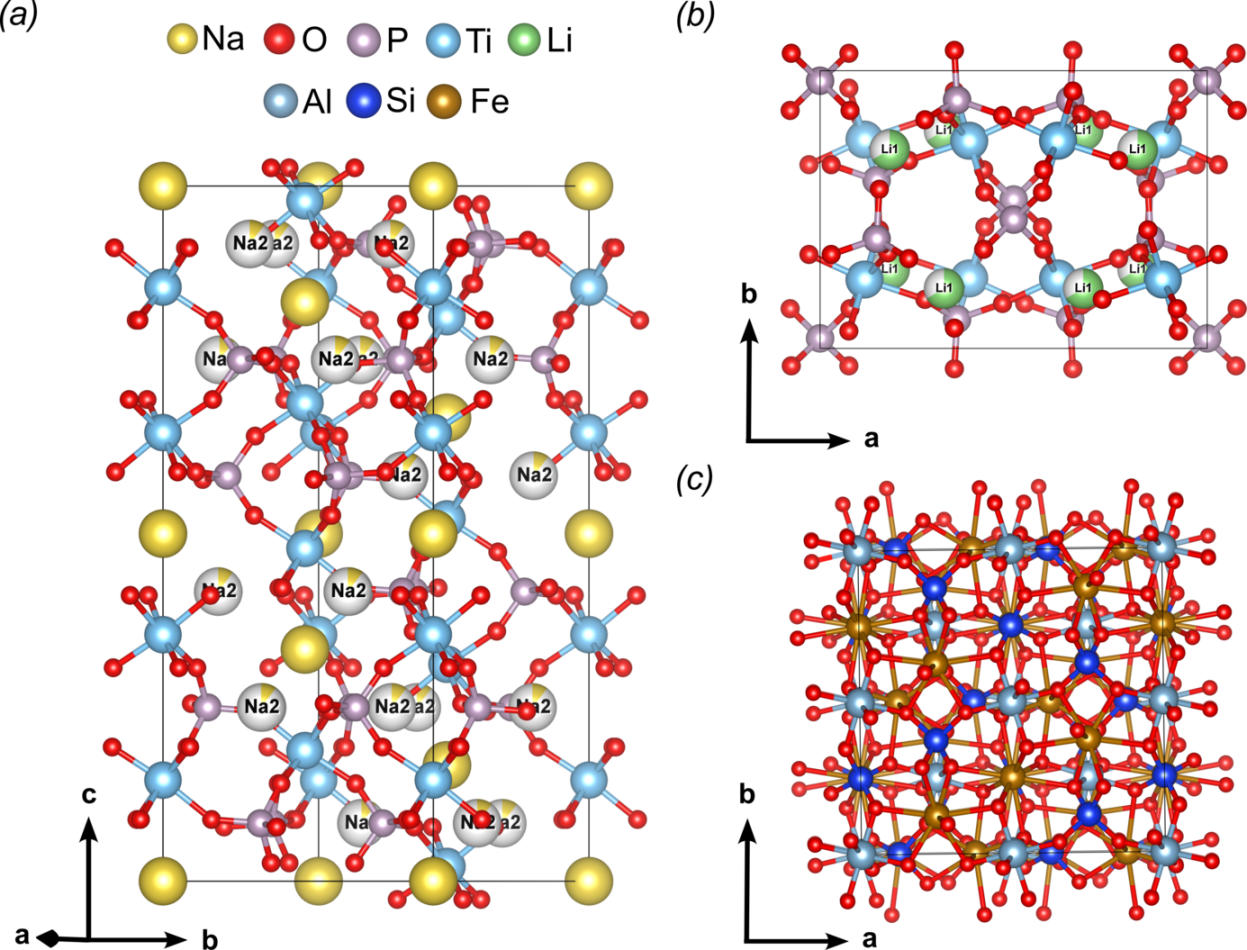
Structural models from 3D ED of (*a*) NATP [Na_1+*x*_Al_*x*_Ti_2−*x*_(PO_4_)_3_], (*b*) LATP [Li_2−*x*_Al_*x*_Ti_2−*x*_(PO_3_)_4_] and (*c*) almandine (Fe_3_Al_2_[SiO_4_]_3_). Partly occupied sites are labelled with the respective atom name.

The extracted intensities for NATP-3, LATP-4 and ALM-2 measured by continuous rotation using the Hitachi microscope at 120 kV were used to obtain initial structure models. All scattering density peaks could be manually assigned to corresponding atom types in the reconstructed potential map of the LATP and almandine phase, while all independent atoms with the exception of the partially occupied *M*3 position, labelled as Na2 in the following, could be assigned in the case of NATP. The correct space groups could be validated. Kinematical refinement of the free structure (atom positions and isotropic displacement parameters) converged to *R*_obs_/*R*_all_ of 23.31%/28.43% (NATP-3), 27.54%/29.80% (LATP-4) and 24.32%/26.60%. (ALM-2). These represent reasonable residuals in the context of electron diffraction, and the kinematically refined models were used as a starting point for the dynamical refinement procedure, from which *R*_obs_/*R*_all_ dropped to 9.71%/12.44% (NATP-3), 11.74%/17.16% (LATP-4) and 7.36%/11.18% (ALM-2). Thereafter, density maxima corresponding to Na2 sites could be located in the difference electrostatic potential (DESP) maps of the NATP refinement. In the LATP refinement, the Li occupancy converged to reasonable values (see Table S4). While partial substitution of Al^3+^ on Ti^4+^ sites should lead to non-stoichiometry, refinement restricting the amount of Al to correspond to the amount of Li did not converge. Thus, the Ti sites were hereafter treated as fully occupied in both models. The derived structure models of the NATP and almandine phases are in accordance with the literature and shown in Fig. 3 ▸. The orthorhombic LATP phase is isostructural with the undoped NASICON-related mixed-valent titanium phosphate phase Li_2_Ti_2_(PO_4_)_3_ (Wang *et al.*, 1993 ▸; Kee *et al.*, 2011 ▸), but had not been observed for LATP before. In the absence of a reduction agent, the lowered Li concentration is a product of charge balancing when introducing Al(III) on Ti(IV) sites in the framework during synthesis, similar to the process of Li insertion described for other lithium metal phosphate species with the general formula Li_2_Ti*M*(PO_4_)_3_ (*M* = Fe, Cr) (Patoux *et al.*, 2004 ▸). The presence of Al was verified by EDX analysis (see Fig. S1), and it was shown that the orthorhombic phase also crystallizes in the *Pbcn* space group. The relatively high *R* values for NATP-3 and LATP-4 after dynamical refinement could be explained by the following reasons related to the experimental setup: (i) longer detector read-out times lead to an only incomplete integration of the rocking curves (see Fig. S2); and (ii) lower acceleration voltages favour dynamical and inelastic scattering (Gholam & Hadermann, 2024 ▸). All other experiments were therefore conducted on another microscope and camera setup with negligible read-out time and at higher acceleration voltage.

### 4.3. Stepwise static tilt

In stepwise static tilt protocols, the width of the missing wedge in the reconstructed diffraction space is a function of the increment used (see Fig. S2), while in the case of continuous rotation the missing data are mainly dependent on the detector read-out times. The effect of the incomplete integration was investigated for the NATP-1 crystal. 3D ED data collected in tilt increments of 0.1° resulted in an output of 751 diffraction patterns, and the extracted reflection intensities were used for dynamical refinement against the structure model derived in Section 4.2, in a procedure similar to the one described in the supporting information in Sections S1 and S2. The refinement converged to *R*_obs_/*R*_all_ = 7.24%/7.99% for *n*_obs_/*n*_all_ = 6309/7892 reflections. While a similar number of reflections were recorded, the incomplete integration of the reflections’ rocking curves led to worse reflection statistics than in the case of continuously measured data (see Tables 2 ▸ and 3 ▸). To analyse how finely the diffraction space has to be sampled, the extracted intensities using every second (0.2°), third (0.3°) and fourth (0.4°) frame were used in the dynamical refinement procedure. Data recorded with a theoretical tilt step of 0.3° already led to strong worsening of the overall quality of the structure model and the residual factors converged to *R*_obs_/*R*_all_ = 13.35%/15.56% for *n*_obs_/*n*_all_ = 6264/9503 reflections.

With an incremental width of 0.4° the residual factors were too high to speak of a model of acceptable quality (see Table S5). When trying to solve the structure *ab initio* via charge flipping or direct methods with data recorded at theoretical tilt steps of 0.3° and 0.4°, no reasonable starting model could be reconstructed from the electrostatic potential map. By averaging a larger number of diffraction patterns, not only the data volume but also the processing time can be greatly reduced. Further, Gaussian noise in the patterns is minimized, in our case revealing detector artefacts hardly visible before (see Fig. S5). When averaging frames, two main effects limit the maximum averaged frame width of such a composite image: ideally the centres of reflections should remain fixed during the tilt, but small pattern shifts occur when shifting the beam because of the crystal tracking, and thus continuous-rotation protocols rarely exceed a tilt increment of 1°. Moreover, the increase in the width of averaged frames results in a higher probability of reflection overlap. Different averaged frame widths were analysed for their potential for structure determination and an average frame width of 1.5° by merging 15 frames provided a good compromise and allowed for a stable unit-cell determination and reflection indexing. The extracted reflections were used for dynamical refinement against the structure model, yielding very similar figures of merit to the non-averaged data (see Table 2 ▸), while greatly speeding up the data reduction steps. A peak search procedure performed on the same computer system at same binning levels for 751 (unmerged) and 50 frames (merged) took 182 s and 10 s, respectively, the execution time thus scaling almost linearly with the number of frames.

### 4.4. Precession versus continuous rotation

3D ED data for the NATP-1, NATP-2, LATP-1, LATP-2 and LATP-3 crystals were collected using both precession-assisted and continuous-rotation methods (the parameters are listed in Table S1). Kinematical refinement against precession data consistently converged to lower *R* values (see Fig. S6), proving to be more suitable as input for kinematical refinement procedures, as described by Palatinus *et al.* (2013 ▸). This trend persisted for the dynamical refinement models, where with the exception of LATP-2 all data sets measured by precession yielded superior refinement statistics. Noteworthy are the *R* values of the NATP-1 crystal measured by precession, where *R*_obs_ drops below 5%, achieving SC-XRD accuracy. The dynamical refinement statistics for all crystals measured by precession and continuous rotation are listed in Table 3 ▸ and Table 4 ▸. The Li content of LATP-1, LATP-2 and LATP-3 was derived by the Al/Ti ratio (0.232) determined by EDX elemental analysis, which results in 1.62 equivalence of Ti and therefore 1.38 equivalence of Li. This is in accordance with the stoichiometric amount of Li precursor (1.3) used in the synthesis and the atomic percentages from the refined chemical formula derived from the structure model (see Table S4). The root-mean-squared deviation (RMSD) in atomic positions of all fully occupied atoms in reference to the XRD models were calculated and amounted to significantly small values, while being practically equal for structures determined by precession and continuous rotation (see Table 3 ▸ and Table 4 ▸).

### 4.5. Subtle features in the structure models

**Figure 4:**
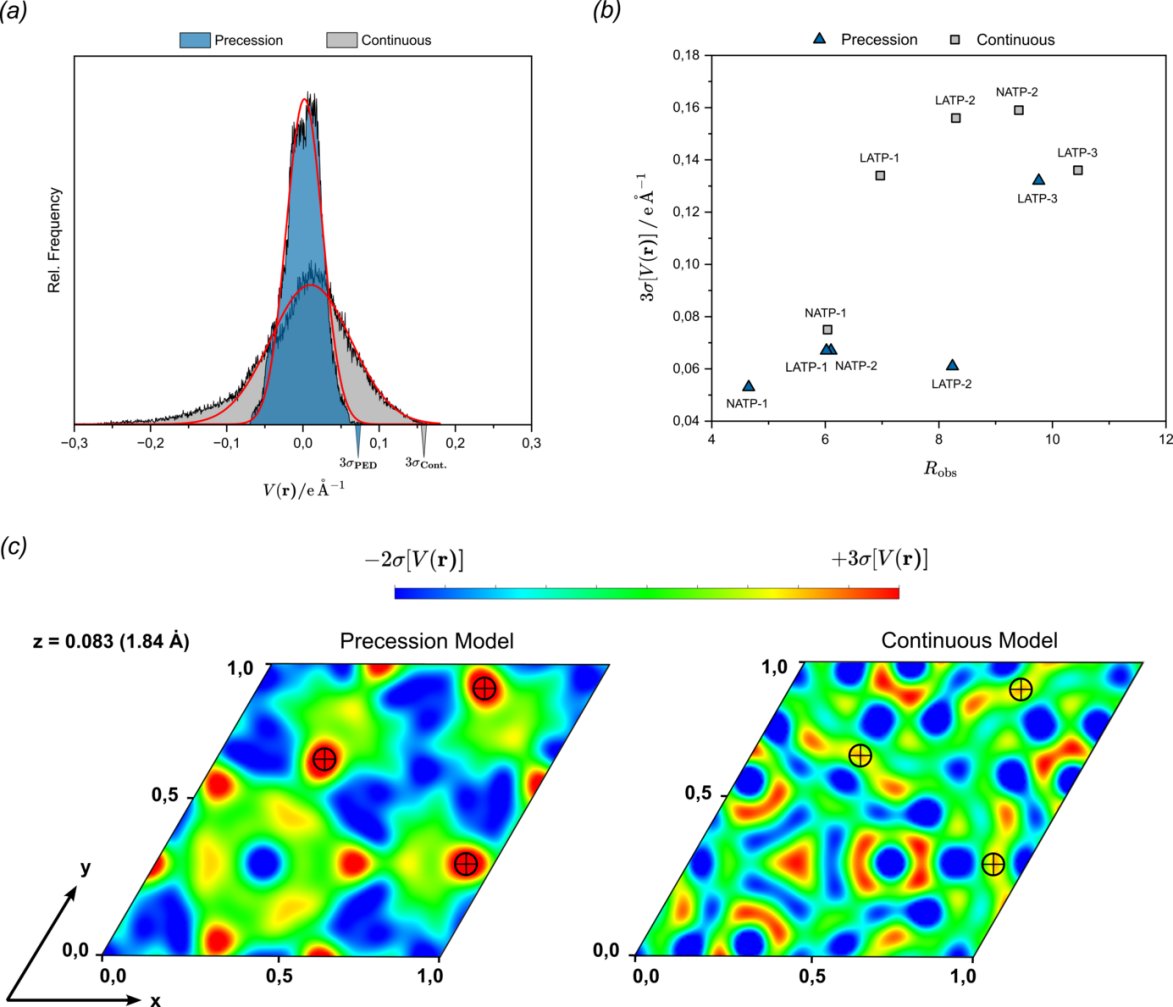
(*a*) Histogram of the residual electrostatic potential calculated from the difference Fourier synthesis of the NATP-2 crystal on the basis of precession and continuous-rotation data. (*b*) 3σ[Δ*V*(**r**)] levels of all five crystals for precession and continuous-rotation data. (*c*) (001) plane at a fractional height of *z* = 0.083 (distance from the origin 1.84 Å) through the Na2-free structure models of the crystal NATP-2. Scattering density peaks corresponding to Na2 atoms are marked by crosshairs.

Fine details were searched for in the crystal structures that contribute little to the total scattering density of the unit cell and are therefore difficult to spot in the DESP maps calculated from the difference Fourier synthesis (*F*_obs_ − *F*_calc_)exp(−2πiφ_calc_). In a nearly complete model, the maps should contain mostly Gaussian noise in the form of an ideally narrow symmetric distribution around zero, and features that are not yet described are said to be of physical interest if their corresponding density peak is approximately three times larger than the standard deviation σ[Δ*V*(**r**)] of the map’s noise. Both precession and continuous-rotation data for the crystals NATP-1, NATP-2, LATP-1, LATP-2 and LATP-3 were used to compute DESP maps and the volumetric data were extracted from the density array. The relative frequency of the residual scattering density was plotted in a histogram for each set of data, and a Gaussian function could be fitted due to being mostly random noise [Fig. 4 ▸(*a*)]. As expected, the crystallographic *R*-factor values correlate with a smaller distribution of the residual density [Fig. 4 ▸(*b*)]. The precession data consistently provide less noisy maps, the level of 3σ[Δ*V*(**r**)] being down to three times smaller than for the continuous-rotation data (listed in Table 3 ▸ and Table 4 ▸). The smaller residual density distribution of the precession maps possibly allows the detection of not-yet-described positive density peaks that are actually not noise.

Owing to the low occupancy of Na2 sites in the NATP phase (<10%), this structural feature was chosen for further evaluation, which is highlighted by the fact that the interpolated peak coordinates could only be determined after dynamical refinement. The Na2-free NATP model was used to compute the DESP maps without further refinement cycles and 2D sections of the DESP maps were plotted at the fractional height of the refined Na2 position, shown in Fig. 4 ▸(*c*). The correctly assigned density peak appeared strongest in the case of precession ED, while being relatively weak in the continuous-rotation map. However, the Na2 position was successfully determined with both methods. The Na2 peak in the continuous-rotation refinement had to be assigned by plausibility considerations regarding the interatomic distance to next neighbour atoms. Such considerations are less straightforward in the study of, for example, small-molecule crystals, where several positions are equally probable due to conformational freedom of the molecular fragments.

### 4.6. Diffuse scattering from Kikuchi lines

**Figure 5:**
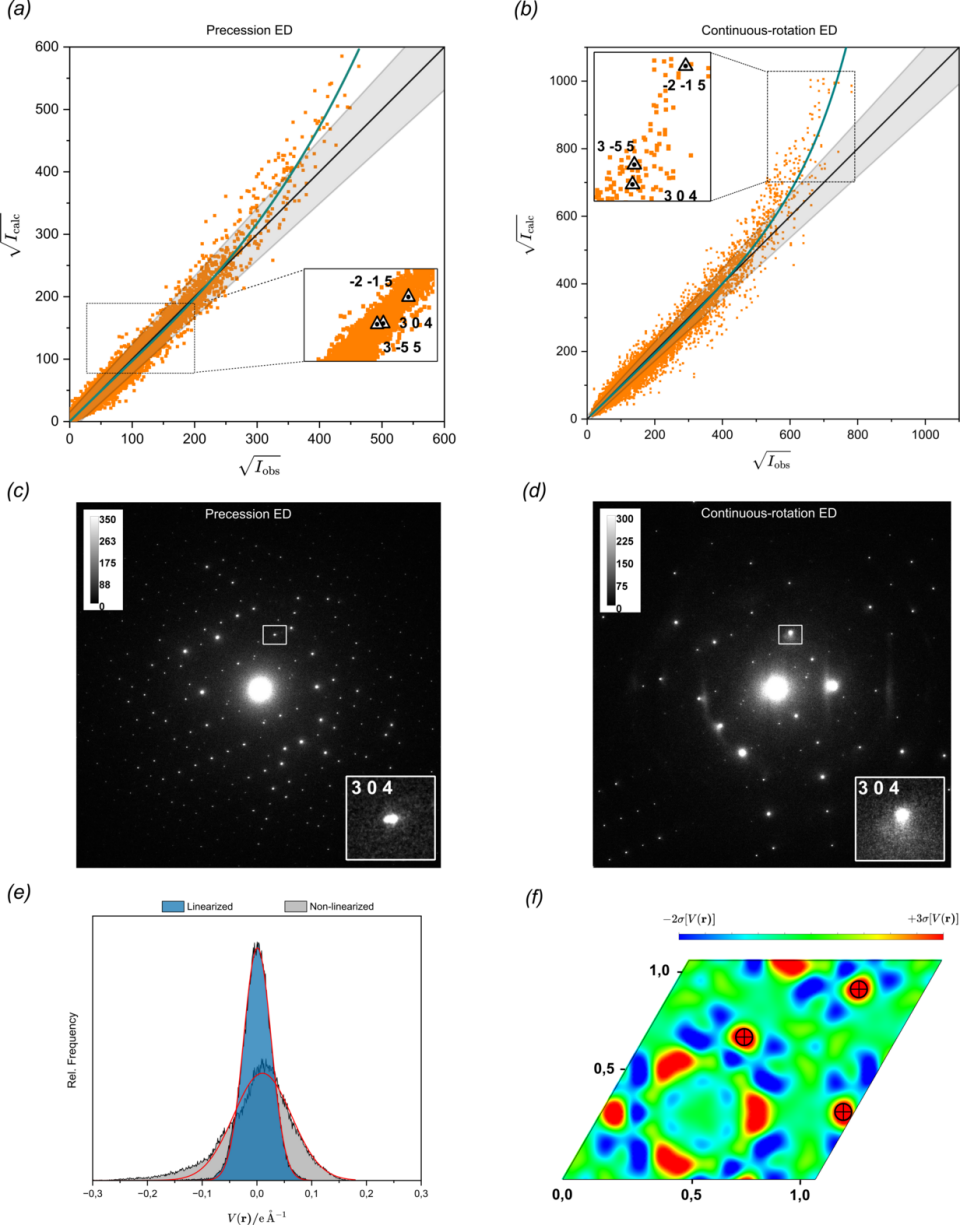
*I*_calc_^1/2^ plotted against *I*_obs_^1/2^ for the thickest crystal, LATP-3, for reflection data acquired by (*a*) precession-assisted and (*b*) continuous-rotation ED. A cubic polynomial (green) was fitted against the reflection data. Reflections lying in a tolerance interval (grey) near the ideal linear condition *I*_obs_^1/2^ = *I*_calc_^1/2^ (black) were treated as an acceptable fit. Some reflections that appear clearly affected by a modulated anisotropic background in the corresponding diffraction patterns are labelled (triangular markers). The non-background-corrected diffraction patterns acquired by (*c*) precession-assisted and (*d*) continuous-rotation ED from the LATP-3 crystal with the outlier reflection 304 as an example shown in a magnified view. The image contrast on both images has been adjusted to be on the same scale for proper comparison. (*g*) Histogram of the residual electrostatic potential of NATP-2 on the basis of linearized and non-linearized continuous-rotation data. (*h*) The (001) plane at a fractional height of *z* = 0.083 through the linearized Na2-free structure model. Scattering density peaks corresponding to Na2 atoms are marked by crosshairs.

To explain the higher accuracy of the structure models and DESP maps derived from precession ED, reflections used in the least-squares refinement were investigated by looking for outliers in the plot of calculated square-root reflection intensities, *I*_calc_^1/2^, against the experimental ones, *I*_obs_^1/2^. A good fit between calculated and experimental data in Fig. 5 ▸(*a*) and (*b*) was expected to result in close scattering around the relation *I*_obs_^1/2^ = *I*_calc_^1/2^, while outliers were those deviating strongly from the ideal linear trend. The intensity distribution due to dynamical scattering effects leads to under-approximation of weak reflections and over-approximation of strong reflections in the kinematical refinement, as described by Midgley & Eggeman (2015 ▸). Surprisingly, a similar trend is observed in the analysed datasets of the ceramic materials when dynamical scattering is considered, with high-intensity reflections still appearing over-approximated to a large extent. While the intensity scale differs between the two plots, which is attributed to the respective integration conditions, the trend can be observed in both the PED and continuous-rotation data, although it is much more pronounced in the latter. A cubic regression model, which best characterizes the deviation from the ideal linear condition, was fitted against the reflection data, and the cubic coefficients were seen to increase in value with respect to the crystal thickness (see Table S6). This trend is amplified for strong reflections recorded by continuous rotation and intensifies drastically as the refined crystal thickness increases (see Fig. S7). The systematic worsening of crystallographic *R* values with respect to the crystal thickness can be attributed to two factors. The first is a stronger random scattering of reflection data due to absorption effects in thicker samples. The variance of the cubic regression function was plotted against different sample thicknesses, clearly declining with increasing crystal size (see Fig. S8). The second is the over-approximation of stronger reflections mentioned above, which we account to diffuse scattering in form of Kikuchi lines, clearly visible in Fig. 5 ▸(*d*) and not considered by the Bloch-wave-based approach used in *Dyngo*.

This aligns well with observations made by Cleverley & Beanland (2023 ▸), who noticed a small fraction of their dynamically refined continuous-rotation data still having a clear dynamical structure in their rocking curves, while also observing Kikuchi lines disturbing the reflections in some patterns. The inspection of pseudo-saturated reflections in Section 4.1 ensures that this trend is solely an experimental quantity and no bias has been introduced during the data processing. The impact of Kikuchi lines on outlier reflections was investigated. If lying in a predefined tolerance interval, reflections were declared as acceptably fitted. The tolerance interval was chosen to correspond to Δ*I*_calc_^1/2^ = 15 ± (*I*_obs_^1/2^ × 0.1), allowing a discrepancy from the linear condition by 10% and anticipating the numerical deviation of weak reflections by some constant. Strong reflections in the plot considered as outliers were traced back to the individual frames, all of them clearly displaying an anisotropic spot profile when collected by continuous-rotation methods and likely hampering the intensity integration by profile fitting. The same reflections were located in the precession data and, as seen in Fig. 5 ▸(*c*) and (*d*), the reflection profiles appear less disturbed (for the respective frames of the other reflections, see Fig. S9). Thicker almandine particles were sought on the grid and recorded by precession ED, and the diffraction patterns displayed prominent Kikuchi lines as well. Fig. S7 shows how the influence of dynamical scattering cannot be neglected for thicker samples even when using beam precession, while for thinner samples the isotropic integration suppresses non-kinematical scattering fairly well.

To quantify the impact of over-approximated outliers, the reflection data were linearized in a procedure similar to the likelihood-based correction applied by Clabbers *et al.*, which was used to account for dynamic scattering in kinematical data (Clabbers *et al.*, 2019 ▸). The linearization was applied for the crystals showing the greatest deviation in their plots and whose diffraction patterns displayed the most prominent Kikuchi lines. The linearized data showed a significant improvement of the *R* factor by Δ*R*_obs_ = −0.99% (NATP-2), Δ*R*_obs_ = −0.38% (LATP-2) and Δ*R*_obs_ = −0.28% (LATP-3). LATP-3 data measured by precession ED were linearized as well and the improvement of Δ*R*_obs_ = −0.1% was less significant. The linearized data of NATP-2 were used to recalculate the relative frequency of the residual scattering density and determine the σ[Δ*V*(**r**)] level of the Gaussian fit function. The 3σ[Δ*V*(**r**)] level of 0.076 e Å^−1^ was almost half that for the non-linearized data. The localization of the Na2 site was re-evaluated by plotting the 2D section at the refined fractional height of Na2 (similar to the procedure in Section 4.5). The noise level was suppressed and allowed a much easier detection of Na2 density peaks than in the non-linearized maps [see. Fig. 5 ▸(*f*)].

## 5. Conclusion

In this work, we presented the application of three-dimensional electron diffraction (3D ED) on three ceramic and mineral phases, two belonging to the NASICON family and finding application as solid-state electrolytes. We employed the most frequently used data collection strategies – precession, continuous rotation and stepwise static ED – on the same crystals maintaining the same experimental conditions. In this context the software *eHermelin* was introduced, allowing for 3D ED experiments in continuous-rotation mode and accounting for many pitfalls often overlooked in routine data acquisition. Merging factors *R*_int_ for data acquired by precession ED were consistently lower than for continuously recorded diffraction data. Incomplete diffraction space coverage appeared to be a problem when working with a slow detector exhibiting long read-out times in continuous-rotation mode, allowing for whole structure determination but leading to worsened refinement statistics. The incomplete integration of the rocking curves is also an inherent problem of stepwise static tilt protocols, where a trade-off between diffraction space coverage and processing time has to be made. We showed that the tilt step increment should be chosen as small as possible (ideally 0.1°) for successful unit-cell and whole-structure determination in such a data-acquisition approach. By averaging multiple frames into a composite image, the data reduction routines can be speeded up and Gaussian noise minimized regardless of the detector model used. Strong dynamical and inelastic scattering are in general the main limiting factors in the quality of the retrieved diffraction patterns. The impact of non-kinematical scattering on continuous-rotation and precession-assisted methods was evaluated in three ways. (i) Crystallographic *R* factors, refinement statistics and the root-mean-square-deviation (RMSD) in atomic positions from reference structures determined by SC-XRD. Both methods yielded accurate structure models, providing almost identical RMSDs. However, data acquired by PED yielded superior refinement statistics, with data from one crystal achieving SC-XRD quality. (ii) For a deeper analysis, the difference electrostatic potential (DESP) maps and their residual noise were inspected. We took advantage of the doped properties of the NATP ceramic, where isomorphous substitution of Al^3+^ against Ti^4+^ leads to incorporation of additional Na^+^ in the crystal structure, which partly (<10%) occupies an intermediate crystallographic site and is difficult to locate in the DESP maps. The observation of this particular dopant in 2D sections of the DESP maps proved to be straightforward in the case of precession ED data, the maps from which consistently displayed lower noise levels. (iii) The difference in the qualities of the retrieved structure models and maps were investigated in terms of the statistics of the individual reflections. The more dynamical nature of data recorded by continuous rotation was demonstrated, clearly showing a systematic over-approximation of strong reflections, which is known for kinematical refinement. This effect could be accounted for by Kikuchi lines, which particularly affect continuously acquired data and correlate with the sample crystal thickness. Reflection spots recorded by precession ED appeared less disturbed, but the trend was observed for thicker crystals as well. By linearizing the extracted reflection intensities, the dynamical nature of the data could be partly compensated for, enabling the visualization of structural features that were previously not statistically significant.

3D ED thus proves itself as suitable tool for the determination of crystal structures of sub-micrometre ceramic particles not accessible by SC-XRD, including the analysis of fine structural features in the crystal framework. Weak scatterers only partly occupying intermediate crystallographic sites were observed with all methods, which paves the way for their analysis in other solid-state electrolytes. The additional sodium ion in the NATP phase was found sitting in the postulated cavity typical for NASICON frameworks and the lithium position in a not-previously-documented ortho­rhombic LATP phase could be freely refined. Nevertheless, the quality of the retrieved structure models and DESP maps strongly depend on an accurate data collection strategy, and we could distinguish several pitfalls in the continuous-rotation method which have to be accounted for. Further, coherent inelastic scattering in form of Kikuchi lines can result in problems for thick ceramic sample crystals and worsen the crystal structure refinement.

## 6. Related literature

The following references are cited in the supporting information: Brázda *et al.* (2022 ▸); Ivanov *et al.* (1980 ▸).

## Supplementary Material

Crystal structure: contains datablock(s) LATP, NATP. DOI: 10.1107/S2052252526003155/of5009sup1.cif

Structure factors: contains datablock(s) LATP. DOI: 10.1107/S2052252526003155/of5009LATPsup2.hkl

Structure factors: contains datablock(s) NATP. DOI: 10.1107/S2052252526003155/of5009NATPsup3.hkl

Details of data processing and structural refinement, and supporting tables and figures. DOI: 10.1107/S2052252526003155/of5009sup4.pdf

Raw data, data reduction inputs and dynamical refinement files for NATP, LATP and almandine: https://doi.org/10.5281/zenodo.17900943

CCDC references: 2540558, 2540559

## Figures and Tables

**Table 1:** Overview of the crystals that were analysed The phases were measured using two different setups, acquisition software and different tilt geometries. Cont. = continuous rotation. Static = stepwise static tilt.

Phase	Label	Acceleration voltage (kV)	Acquisition software	Method
Na_1+*x*_Al_*x*_Ti_2−*x*_(PO_4_)_3_	NATP-1	200	*Fast-ADT*	PED/Cont./Static
	NATP-2	200	*Fast-ADT*	PED/Cont.
	NATP-3	120	*eHermelin*	Cont.
Li_2−*x*_Al_*x*_Ti_2−*x*_(PO_3_)_4_	LATP-1	200	*Fast-ADT*	PED/Cont.
	LATP-2	200	*Fast-ADT*	PED/Cont.
	LATP-3	200	*Fast-ADT*	PED/Cont.
	LATP-4	120	*eHermelin*	Cont.
Fe_3_Al_2_[SiO_4_]_3_	ALM-1	200	*Fast-ADT*	PED
	ALM-2	120	*eHermelin*	Cont.

**Table 2:** Dynamical refinement statistics based on *F*^2^(*h*)/*I*(*h*) for experimental 3D ED data acquired by means of stepwise static tilt of the NATP-1 crystal Reflections with *F*^2^ < 3σ(*F*^2^) were considered unobserved.

	Static (0.1°)	Static with averaged frame width (1.5°)
Completeness (%)	96.8	97.4
*n*_obs_/*n*_all_	6309/7892	2956/6552
*R*_int,obs_/*R*_int,all_ (%)	11.85/13.96	11.47/13.89
*R*_obs_/*R*_all_ (%)	7.24/7.99	6.76/9.98
*wR*_obs_/*wR*_all_ (%)	8.39/8.43	7.68/7.84
GoF_obs_/GoF_all_	4.34/3.91	2.70/1.85
No. of parameters	106	81

**Table 3:** Dynamical refinement statistics based on on *F*^2^(*h*)/*I*(*h*) against experimental 3D ED data acquired by means of precession (PED) and continuous rotation (Cont.) for NATP-1 and NATP-2 crystals Reflections *F*^2^ < 3σ(*F*^2^) were considered unobserved.

	NATP-1		NATP-2	
	PED	Cont.	PED	Cont.
Completeness (%)	99.2	96.2	94.9	95.8
*n*_obs_/*n*_all_	3656/12400	6442/7362	10640/19103	11308/11398
*R*_int,obs_/*R*_int,all_ (%)	8.26/10.58	12.81/15.92	16.33/19.32	12.48/13.58
*R*_obs_/*R*_all_ (%)	4.65/10.36	6.04/6.32	6.10/8.19	9.41/9.42
*wR*_obs_/*wR*_all_ (%)	5.01/5.39	7.06/7.08	6.63/6.81	11.92/11.92
GoF_obs_/GoF_all_	1.60/0.94	3.59/3.37	2.13/1.64	7.92/7.89
No. of parameters	77	105	147	145
RMSD (Å)	0.006	0.006	0.005	0.006
3σ[Δ*V*(**r**)] (e Å^−1^)	0.053	0.075	0.067	0.159

**Table 4:** Dynamical refinement statistics based on *F*^2^(*h*)/*I*(*h*) against experimental 3D ED data acquired by means of precession (PED) and continuous rotation (Cont.) for LATP-1, LATP-2 and LATP-3 crystals Reflections with *F*^2^ < 3σ(*F*^2^) were considered unobserved.

Crystal	LATP-1		LATP-2		LATP-3	
Method	PED	Cont.	PED	Cont.	PED	Cont.
Completeness (%)	85.5	84.3	96.4	96.3	96.4	83.5
*n*_obs_/*n*_all_	13533/32376	18140/20095	22341/33278	16401/16626	20842/38313	20192/20595
*R*_int,obs_/*R*_int,all_ (%)	12.48/13.58	14.25/18.48	14.98/16.81	18.82/28.29	19.21/19.72	31.01/34.82
*R*_obs_/*R*_all_ (%)	6.02/9.14	6.97/7.35	8.24/9.46	8.30/8.31	9.76/12.10	10.45/10.49
*wR*_obs_/*wR*_all_ (%)	6.61/6.81	8.33/8.36	9.39/9.49	10.31/10.31	10.66/10.92	13.02/13.02
GoF_obs_/GoF_all_	2.34/1.56	4.41/4.20	3.16/2.63	6.43/6.39	2.93/2.33	7.13/7.07
No. of parameters	168	166	204	202	184	182
RMSD (Å)	0.020	0.020	0.018	0.019	0.012	0.014
3σ[Δ*V*(**r**)] (e Å^−1^)	0.067	0.134	0.061	0.156	0.132	0.136

## Data Availability

All data needed to evaluate the conclusions in the paper are part of the supporting materials or can be downloaded. The raw image files, data reduction files (*PETS2* files) and dynamical refinement files (*JANA2020* files) for all compounds used in the study are available at https://doi.org/10.5281/zenodo.17900943, a repository hosted by Zenodo.
