# Supplementary material for: Benchmarking 3D electron diffraction strategies for ceramics
Source: IUCrJ. 2026 Apr 27;13(Pt 3):291–303. doi: 10.1107/S2052252526003155 (PMC13134496; doi:10.1107/S2052252526003155)
Supplement: Supplementary file 4 [file m-13-00291-sup4.pdf]

IUCrJ

Volume 13 (2026)

Supporting information for article:

Benchmarking 3D electron diffraction strategies for ceramics

Yann Schmitt, Sergi Plana-Ruiz and Yaşar Krysiak

S1. Procedure of 3D ED data processing

In a peak hunt reflections and the diffraction pattern centres were located, and the signal-to-noise adjusted to a reasonable level. The position of the tilt axis with respect to the horizontal image axis was refined. On basis of the calculated difference vector space a set of unit cell parameter could be determined. The effective camera length was calibrated with the unit cell parameters acquired by Powder-XRD and the size of a pixel in reciprocal angstroms rescaled for subsequent data reduction (Brázda *et al.*, 2022). The *PETS2* file was saved and the orientation matrix derived from another ED experiment performed on the same sample crystal copied into the output file. In the new orientation frame the unit cell as well as optical distortions (in-plane rotation, barrel–pincushion, spiral, elliptical; for PED also parabolic) were refined restricting the cell parameters to the correct crystal symmetry. An initial frame-based orientation refinement was performed against uniform intensities. A moving average was chosen to reduce noise, but without wrongly averaging out fluctuations which were indeed a consequence of the specimen motion. Rocking-curve width and apparent mosaicity were set to 0.003 $Å^{-1}$ and 0.01°, enabling a first complete integration despite too broad curves. The precession angle was correctly adjusted and the integration finalized with the fit profile method. The frame scaling was chosen to correspond to an appropriate tilt range (3-5°) with a correlation factor of 0.5 in the correct Laue group (mmm and -3m), while omitting saturated reflections. As the target function minimized in the least square optimisation of the frames orientation provides more accurate results against a set of integrated intensities, the step has to be repeated. The refined orientation parameters in turn lead to an improved positioning of the integration boxes, which allows for a more accurate rocking curve fitting. As the extracted intensities in turn improve all subsequent routines, an iterative processing cycle is entered. The signal-to-noise parameter in the rocking curve fitting, used to select the reflections involved in the rocking curve calculation, was reduced till calculated and experimental rocking curves displayed the best match. The intensity integration was monitored by verifying the merging R-factors over all resolution shells after every processing cycle as well as the refined error model. The space groups $R\bar{3}c$ and $Pbcn$ were confirmed by checking the extincted reflections in the reconstructed reciprocal space sections.

S2. Procedure of structure refinement

The procedure of structure refinement is laid out exemplary for the crystals NATP-1 and LATP-1. Other refinements were conducted in a similar fashion. When measuring using both precession-assisted and continuous-rotation methods, the data set acquired by precession was used to establish a correct structure model, which was used to refine against continuously recorded data. The extracted reflection intensities of crystal NATP-1 and LATP-1 were used to solve the structure *ab-initio* either via charge flipping or direct methods in the space group $Pbcn$. The correct space group was revalidated based on the reconstructed scattering density maps. All scattering density peaks could be

manually assigned to corresponding atom types in the initial LATP model, while all independent atoms with exceptions of the Na2 atoms could be assigned in the initial NATP model. The starting models were refined kinematically without any restrains using isotropic atomic displacement parameters (ADPs) against the kinematical reflection intensities. Kinematical refinement using anisotropic ADPs resulted in an already stable structure model for LATP, while for NATP all ADPs with exception of the Phosphor atom converged to reasonable values. The kinematic models were used as basis for dynamical refinement and all structure parameters were set fixed to first optimize the average crystal thickness and the frame scale factors, initially resulting in a drop of R_{obs} under 10 % for both phases. For all data-sets the number of orientations for the Bloch-wave calculation was gradually increased until the refinement results converged to consistent values. The U_{iso} of both the Li atom in LATP and the Na2 atom in NATP were set fixed at a value of 0.02 according to literature XRD performed at room temperature (Kee *et al.*, 2011; Ivanov *et al.*, 1980). The dynamical refinement of the LATP model with free parameters (xyz and ADPs) against the precession data yielded a R_{obs}-value of 6.96 %, decreasing after an additional orientation optimization down to $R_{\mathrm{obs}} = 6.61$ %. After dynamical refinement of the kinematical NATP model against PED data the missing Na2 atoms could be localized in the difference electrostatic potential (DESP) maps and the phosphor ADPs converged to reasonable positive values. The structure parameters were set free with exception of the displacement parameter of the partly occupied atoms. To account for the Al^{3+}-occupancy on shared Ti^{4+} sites, refinement with split positions was performed while restricting the occupancy of Al^{3+} to be equal to Na2. Because the refinement did not converge, solely Ti atoms were used in the structure model. After the last refinement setting all parameters free (with exception of U_{iso} of Na2) an additional orientation optimization was performed and the final R_{obs} converged to 4.65 %.

Table S1 Experimental parameters for the data collection by continuous-rotation and precession ED at the JEOL F200 ColdFEG TEM.

	NATP-1	NATP-2	LATP-1	LATP-2	LATP-3
Radiation type			Electrons, 200 kV		
Wavelength (pm)			2.508		
Temperature (K)			295.15		
Tilt range (°)	[-35, +40]	[-50, 60]	[-60, +25]	[-60, 60]	[-40, 60]
Step size (°) (PED / Cont.)	1 / 0.25	1 / 0.25	1 / 0.25	1 / 0.25	1 / 0.25
Precession angle (°)			1		
Detector Binning			2		
Exp. time per frame (s)	0.4/1	0.4/1	0.16/0.5	0.16/0.5	0.16/0.5
No. frames (PED / Cont.)	76 / 301	111 / 441	86 / 341	121 / 481	101 / 401

Table S2 Experimental parameters for the data collection by stepwise static rotation ED at the JEOL F200 ColdFEG TEM.

	NATP-1
Radiation type	Electrons, 200 kV
Wavelength (pm)	2.508
Temperature (K)	295.15
Tilt range (°)	[-35, +40]
Step size (°)	0.1
Exp. time per frame (s)	1
No. frames	751

Table S3 Experimental parameters for the data collection by continuous-rotation ED at the Hitachi HT7800 TEM.

	NATP-3	LATP-3	ALM-2
Radiation type		Electrons, 120 kV	
Wavelength (pm)		3.349	
Temperature (K)		295.15	
Tilt range (°)	[-50, +50]	[-50, +50]	[-40, +50]
Step size (°)	1	1	0.5
Detector Binning		2	
Exp. time per frame (s)	1.08	1.08	0.74
No. frames	101	101	181

Table S4 Crystallographic data and parameters of the dynamical refinement procedure in *JANA2020*. Ti and Al amount in the empirical formula were calculated on basis of the Li amount.

**NATP-1**		
**Method**	**PED**	**Continuous-rotation**
Empirical Formula	$Na_{1.33}Al_{0.33}Ti_{1.67}(PO_4)_3$	$Na_{1.33}Al_{0.33}Ti_{1.67}(PO_4)_3$
a / b / c (Å)	8.4822 / 8.4822 / 21.734	8.482 / 8.482 / 21.687
α / β / γ (°)	90 / 90 / 120	90 / 90 / 120
V (Å^3)	1354.3	1351.4
Z	6	6
OVF (N_F/N_O)	1 / 1	8 / 4
g(max)	2	2
RSg(max)	0.8	0.8
SCA diameter	2.1	2.1
DSg(min)	0.0017	0.0017
No. integration steps	120	30
Average crystal thickness (Å)	537	554
**NATP-2**		
Empirical Formula	$Na_{1.21}Al_{0.21}Ti_{1.79}(PO_4)_3$	$Na_{1.21}Al_{0.21}Ti_{1.79}(PO_4)_3$
a / b / c (Å)	8.505 / 8.505 / 21.757	8.497 / 8.497 / 21.774
α / β / γ (°)	90 / 90 / 120	90 / 90 / 120
V (Å^3)	1363.1	1361.5
OVF (N_F/N_O)	1 / 1	8 / 4
g(max)	2	2

RSg(max)	0.8	0.8
SCA diameter	10	10
DSg(min)	0.0017	0.0017
No. integration steps	240	180
Average crystal thickness (Å)	1617	1976
**NATP-3**		
Empirical Formula	$Na_{1.28}Al_{0.28}Ti_{1.72}(PO_4)_3$	
a / b / c (Å)	8.499 / 8.499 / 21.795	
α / β / γ (°)	90 / 90 / 120	
V (Å^3)	1363.7	
OVF (N_F/N_O)	1 / 1	
g(max)	2	
RSg(max)	0.66	
SCA diameter	10	
DSg(min)	0.0017	
No. integration steps	60	
Average crystal thickness (Å)	1446	
**LATP-1**		
Chemical Formula	$Li_{1.60}Al_{0.4}Ti_{1.6}(PO_3)_4$	$Li_{1.33}Al_{0.67}Ti_{1.33}(PO_3)_4$
a / b / c (Å)	11.961 / 8.629 / 8.673	11.985 / 8.599 / 8.688
α / β / γ (°)	90 / 90 / 90	90 / 90 / 90
V (Å^3)	895.1	895.4
Z	4	4
OVF (N_F/N_O)	1 / 1	8 / 4
g(max)	2	2
RSg(max)	0.8	0.8
SCA diameter	2.1	2.1
DSg(min)	0.0017	0.0017
No. integration steps	144	60
Average crystal thickness (Å)	987	1054
**LATP-2**		
Empirical Formula	$Li_{1.58}Al_{0.42}Ti_{1.58}(PO_4)_3$	$Li_{1.48}Al_{0.52}Ti_{1.48}(PO_4)_3$
a / b / c (Å)	11.962 / 8.582 / 8.647	11.974 / 8.587 / 8.658
α / β / γ (°)	90 / 90 / 90	90 / 90 / 90
V (Å^3)	887.7	890.1
OVF (N_F/N_O)	1 / 1	8 / 4
g(max)	1.8	1.8
RSg(max)	0.8	0.8
SCA diameter	2.1	10

DSg(min)	0.0017	0.0017
No. integration steps	210	90
Average crystal thickness (Å)	932	1051
**LATP-3**		
Empirical Formula	$Li_{1.75}Al_{0.25}Ti_{1.75}(PO_4)_3$	$Li_{1.77}Al_{0.23}Ti_{1.77}(PO_4)_3$
a / b / c (Å)	12.033 / 8.609 / 8.673	12.051 / 8.608 / 8.682
α / β / γ (°)	90 / 90 / 90	90 / 90 / 90
V (Å^3)	898.5	900.6
OVF (N_F/N_O)	1 / 1	8 / 4
g(max)	2	2
RSg(max)	0.8	0.8
SCA diameter	10	10
DSg(min)	0.0017	0.0017
No. integration steps	360	180
Average crystal thickness (Å)	2582	2291
**LATP-4**		
Empirical Formula		$Li_{1.14}Al_{0.86}Ti_{1.14}(PO_4)_3$
a / b / c (Å)		12.059 / 8.654 / 8.725
α / β / γ (°)		90 / 90 / 90
V (Å^3)		910.4
OVF (N_F/N_O)		3 / 2
g(max)		1.7
RSg(max)		0.8
SCA diameter		2.1
DSg(min)		0.0017
No. integration steps		120
Average crystal thickness (Å)		1511
**ALM-1**		
Empirical Formula	$Fe_3Al_2(SiO_4)_3$	
a / b / c (Å)	11.605 / 11.605 / 11.605	
α / β / γ (°)	90 / 90 / 90	
V (Å^3)	1562.7	
OVF (N_F/N_O)	1 / 1	
g(max)	2	
RSg(max)	0.8	
SCA diameter	10	
DSg(min)	0	
No. integration steps	240	
Average crystal thickness (Å)	1933	

ALM-2	
Empirical Formula	$Fe_3Al_2(SiO_4)_3$
a / b / c (Å)	11.609 / 11.609 / 11.609
α / β / γ (°)	90 / 90 / 90
V (Å^3)	1564.8
Z	8
OVF (N_F/N_O)	4 / 2
g(max)	2
RSg(max)	0.66
SCA diameter	2.1
DSg(min)	0.0017
No. integration steps	60
Average crystal thickness (Å)	987

Table S5 Dynamical refinement statistics against experimental 3D ED data done on $F^2(\boldsymbol{h})$ / $I(\boldsymbol{h})$ acquired in a stepwise tilt fashion with tilt increments of 0.2°, 0.3° and 0.4° for the NATP-01 crystal (reflections $F^2 < 3\sigma(F^2)$ were considered unobserved).

Method	Static 0.2°	Static 0.3°	Static 0.4°
a / b / c (Å)	8.531 / 8.531 / 21.871	8.596 / 8.596 / 22.031	8.597 / 8.597 / 22.025
V (Å^3)	1377.5	1409.5	1409.6
Completeness (%)	97.5	96.0	92.5
$R_{\mathrm{Int,Obs}}$ / $R_{\mathrm{Int,All}}$ (%)	13.00 / 15.41	14.78 / 18.95	16.50 / 22.83
n_{Obs} / n_{All}	5776 / 7905	6264 / 9503	4175 / 6770
R_{Obs} / R_{All} (%)	9.28 / 10.44	13.35 / 15.46	17.21 / 19.93
wR_{Obs}/ wR_{All} (%)	10.71 / 10.77	15.56 / 15.66	20.23 / 20.36
$\mathrm{GoF_{Obs}}$ / $\mathrm{GoF_{All}}$	5.02 / 4.32	6.80 / 5.63	8.53 / 6.78
No. of refined parameters	105	113	93

Table S6 Terms of the polynomial $f(x) = \beta_1 x + \beta_2 x^2 + \beta_3 x^3$ with fixed interception at $f(x) = 0$ used to model the reflection data.

Crystal	β_1	β_2 / 10^{-4}	β_3 / 10^{-6}
NATP 1 Cont.	1.027 ± 0.003	-6.3 ± 0.4	2.3 ± 0.1
NATP 2 Cont.	1.056 ± 0.004	0	3.00 ± 0.06
LATP 01 Cont.	1.025 ± 0.002	-6.9 ± 0.2	2.59 ± 0.05
LATP 02 Cont.	1.030 ± 0.003	-7.1 ± 0.3	2.16 ± 0.06
LATP 03 Cont.	0.974 ± 0.003	-2.9 ± 0.4	3.0 ± 0.1

Table S7 Crystallographic data and dynamical refinement statistics against experimental data done on $F^2(\boldsymbol{h})$ / $I(\boldsymbol{h})$ acquired by means of continuous-rotation of the NATP-3, LATP-4 and ALM-2 phase measured with *eHermelin*.

**NATP-3**	
Completeness (%)	93.3
n_{Obs} / n_{All} (< $3\sigma(F^2)$)	2767 / 8404
$R_{\mathrm{Int,Obs}}$ / $R_{\mathrm{Int,All}}$ (%)	19.67 / 26.83
R_{Obs} / R_{All} (%)	7.95 / 12.12
wR_{Obs}/ wR_{All} (%)	16.02 / 17.11
$\mathrm{GoF_{Obs}}$ / $\mathrm{GoF_{All}}$	1.96 / 1.22
No. of refined parameters	96
**LATP-4**	
Completeness (%)	92.9
n_{Obs} / n_{All} (< $3\sigma(F^2)$)	2382 / 8521
$R_{\mathrm{Int,Obs}}$ / $R_{\mathrm{Int,All}}$ (%)	19.72 / 28.53
R_{Obs} / R_{All} (%)	11.74 / 17.16
wR_{Obs}/ wR_{All} (%)	12.57 / 13.37
$\mathrm{GoF_{Obs}}$ / $\mathrm{GoF_{All}}$	2.56 / 1.47
No. of refined parameters	86

**ALM-2**	
Completeness (%)	86.3
n_{Obs} / n_{All} (< $3\sigma(F^2)$)	2470 / 6644
$R_{\mathrm{Int,Obs}}$ / $R_{\mathrm{Int,All}}$ (%)	32.22 / 51.18
R_{Obs} / R_{All} (%)	7.36 / 11.18
wR_{Obs}/ wR_{All} (%)	7.44 / 7.75
$\mathrm{GoF}_{\mathrm{Obs}}$ / $\mathrm{GoF}_{\mathrm{All}}$	2.3 / 1.47
No. of refined parameters	138

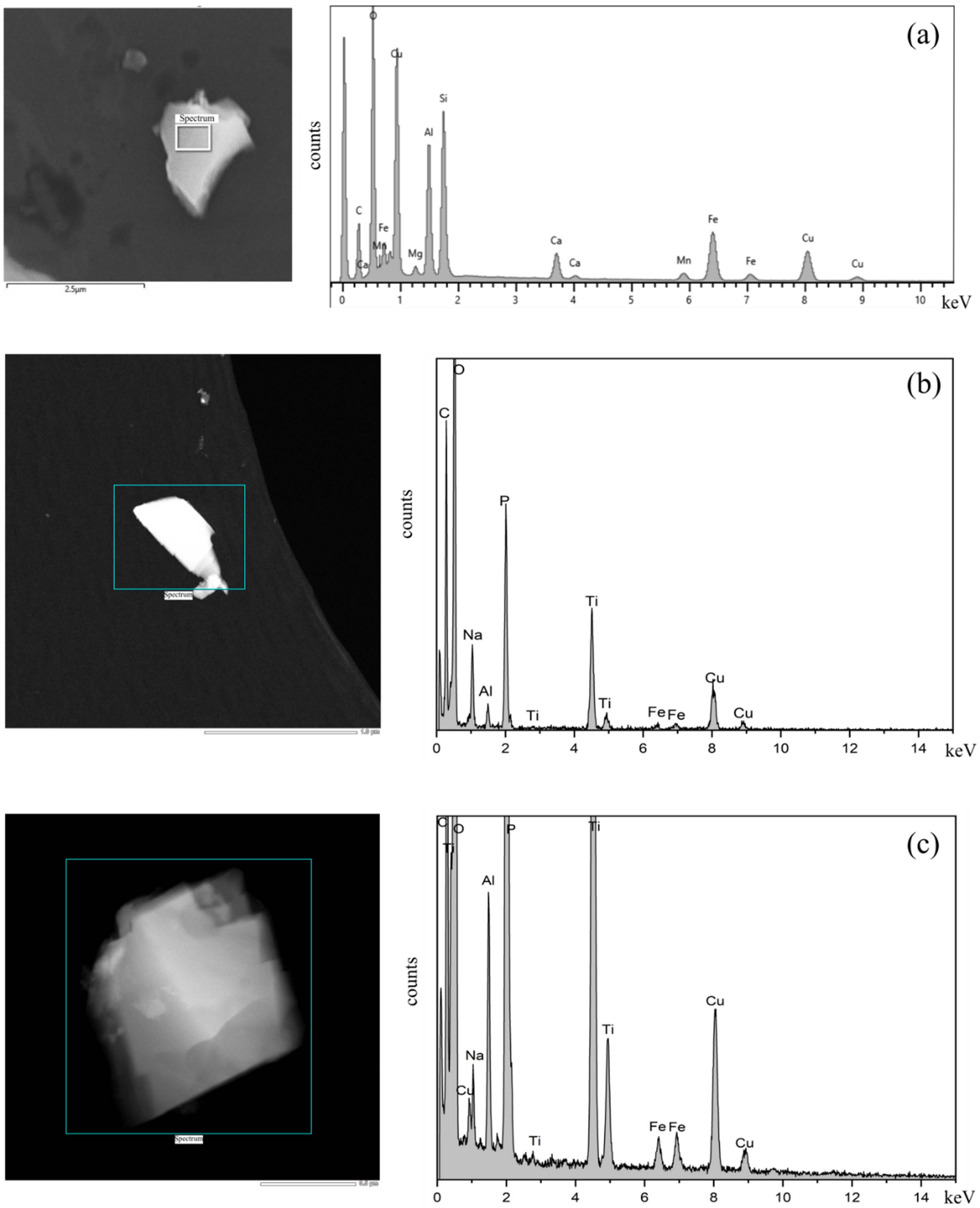

Figure S1 EDX spectra and micrographs of the (a) almandine, (b) NATP and (c) LATP phase.

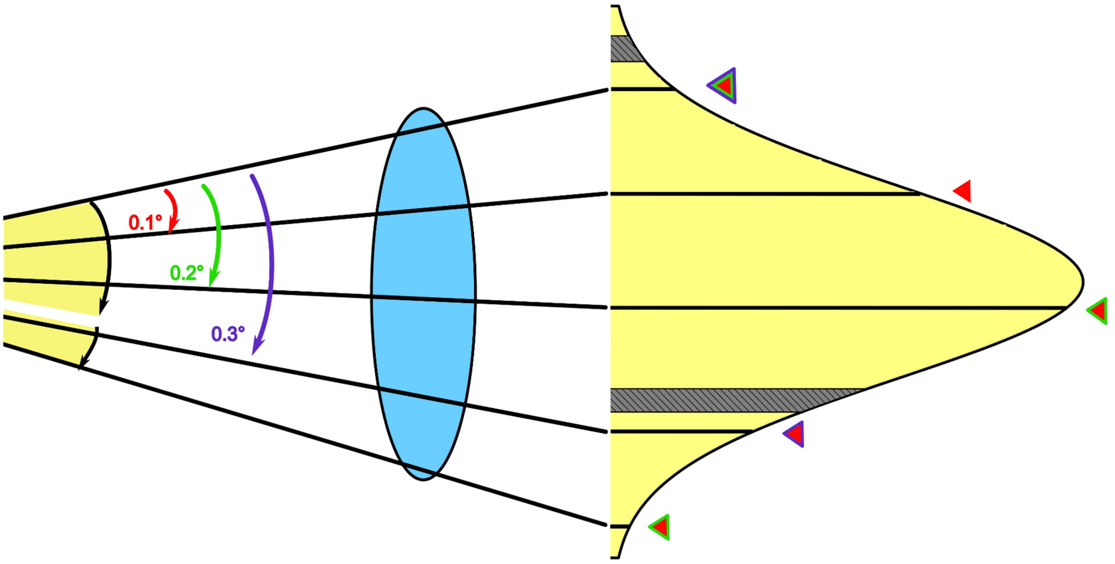

Figure S2 Due to the finite diffraction volume of the sample and factors such as mosaicity, reflection intensities are not confined to singular points in reciprocal space but are instead distributed according to a function of the excitation error. Continuous-rotation data acquisition enables numerical integration perpendicular to the tilt axis across the majority of the reflection-intensity profile (yellow area), while short detector read-out times are essential to minimize loss of information (grey area). In contrast, stepwise tilting permits only incremental integration and poses the risk of not exciting the reflection at all if the tilt increment is chosen too large. The coloured triangles indicate which tilt-step widths correspond to the respective integration point.

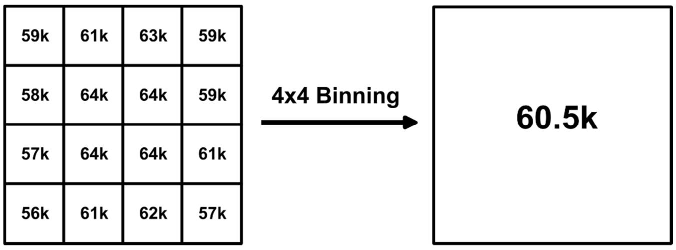

Figure S3 Schematic representation of pseudo non-saturated pixels: due to the averaging of pixels, which are clearly saturated (the bit depth of the used detector being about 64000), the binned pixel appears not saturated and is thus not filtered out during the data reduction routine.

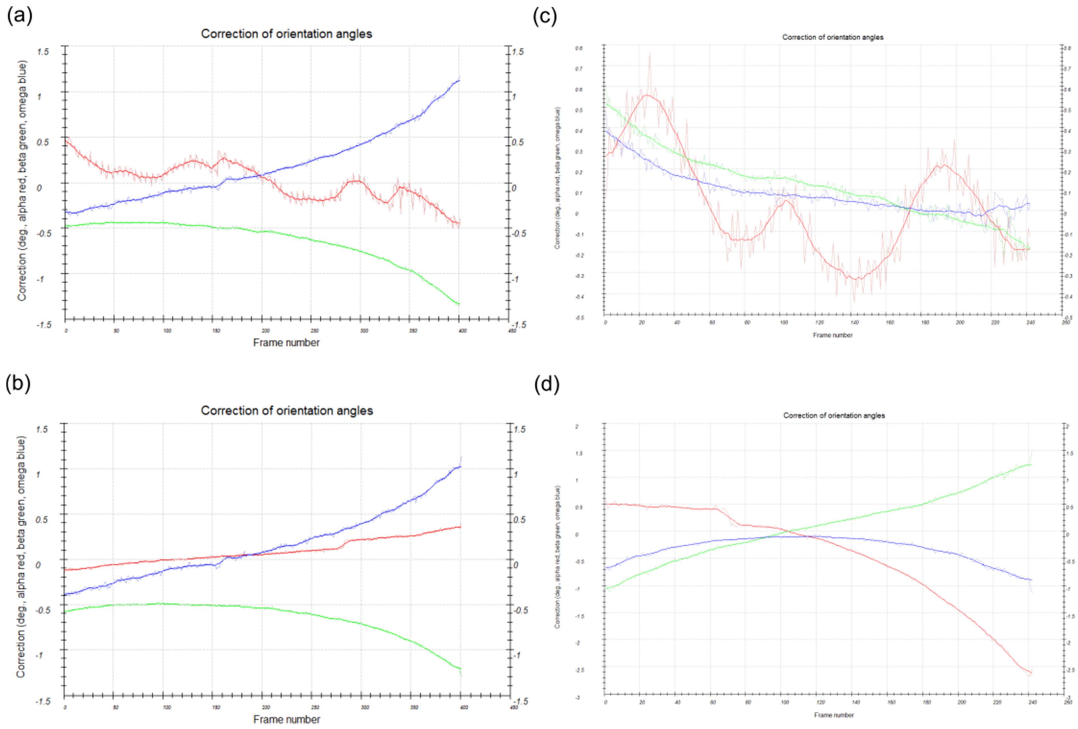

Figure S4 Tilt correction plots for the three goniometer angles α (red), β (green) and ω (blue) for continuously recorded ED data of the NATP-1 (left) and Almandine crystal (right). The plots were recorded with a JEOL analytical tomography holder with the (a) retrieved tilt angles during the experiment and (b) calculated tilt angles. Opposed are the plots recorded with a Hitachi HT7800 single-tilt holder for the (c) live retrieved tilt angles during the experiment and (d) calculated tilt angles.

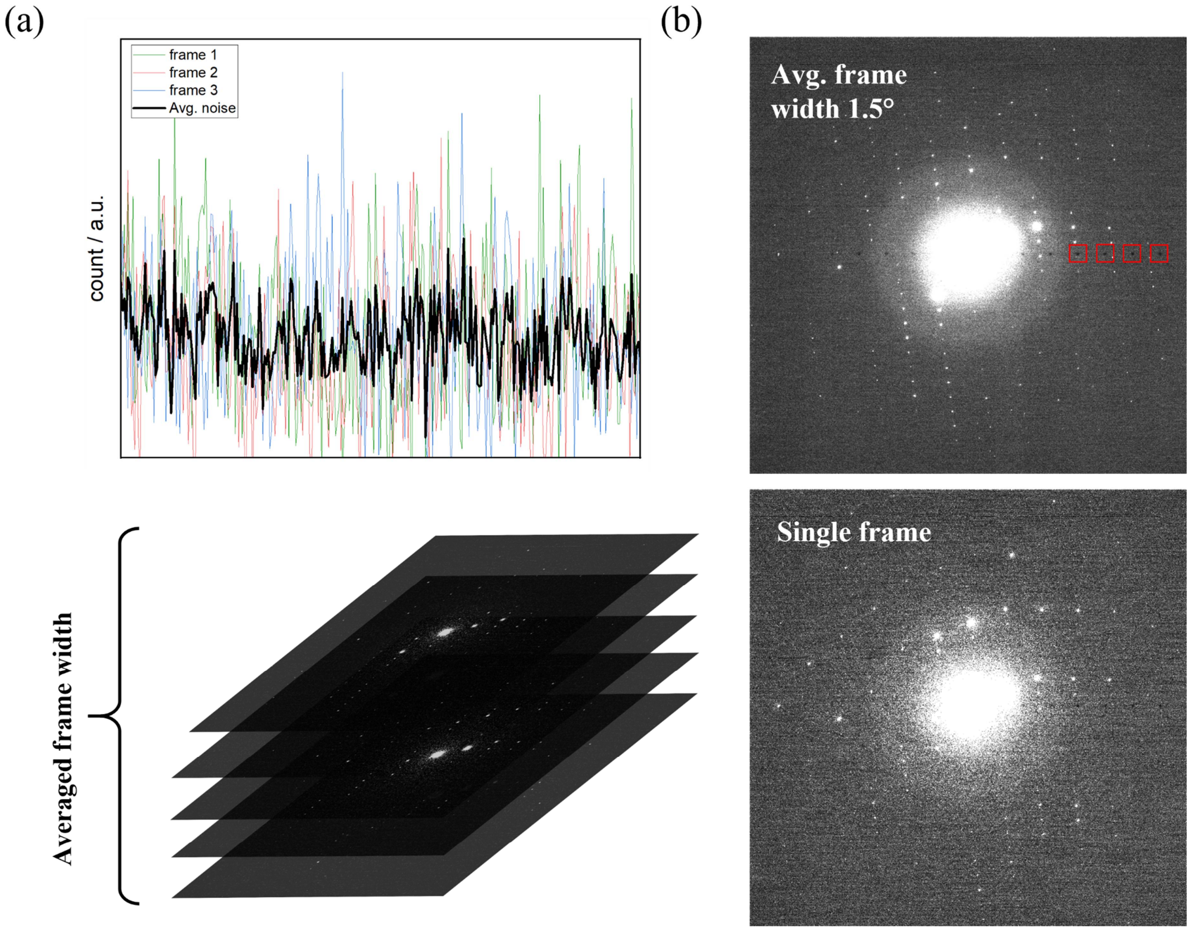

Figure S5 (a) By averaging diffraction patterns into a composite image, Gaussian noise in the micrograph can be reduced, as seen for the line profile of three different frames. (b) The unprocessed images are displayed at the same white value. The averaged frame appears less noisy, and detector artefacts became better visible, some marked in red.

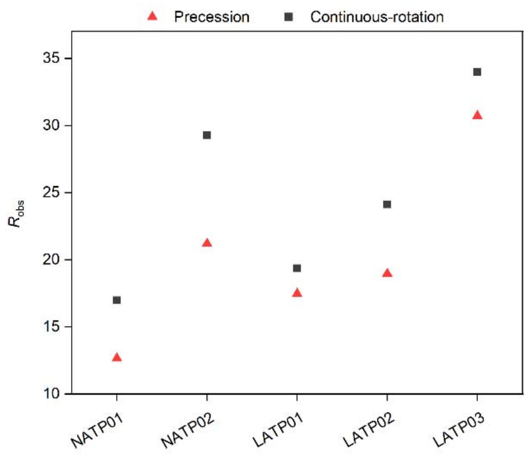

Figure S6 R_{obs}-values of the kinematical refinement plotted for crystals NATP-1, NATP-2, LATP-1, LATP-2 and LATP-3.

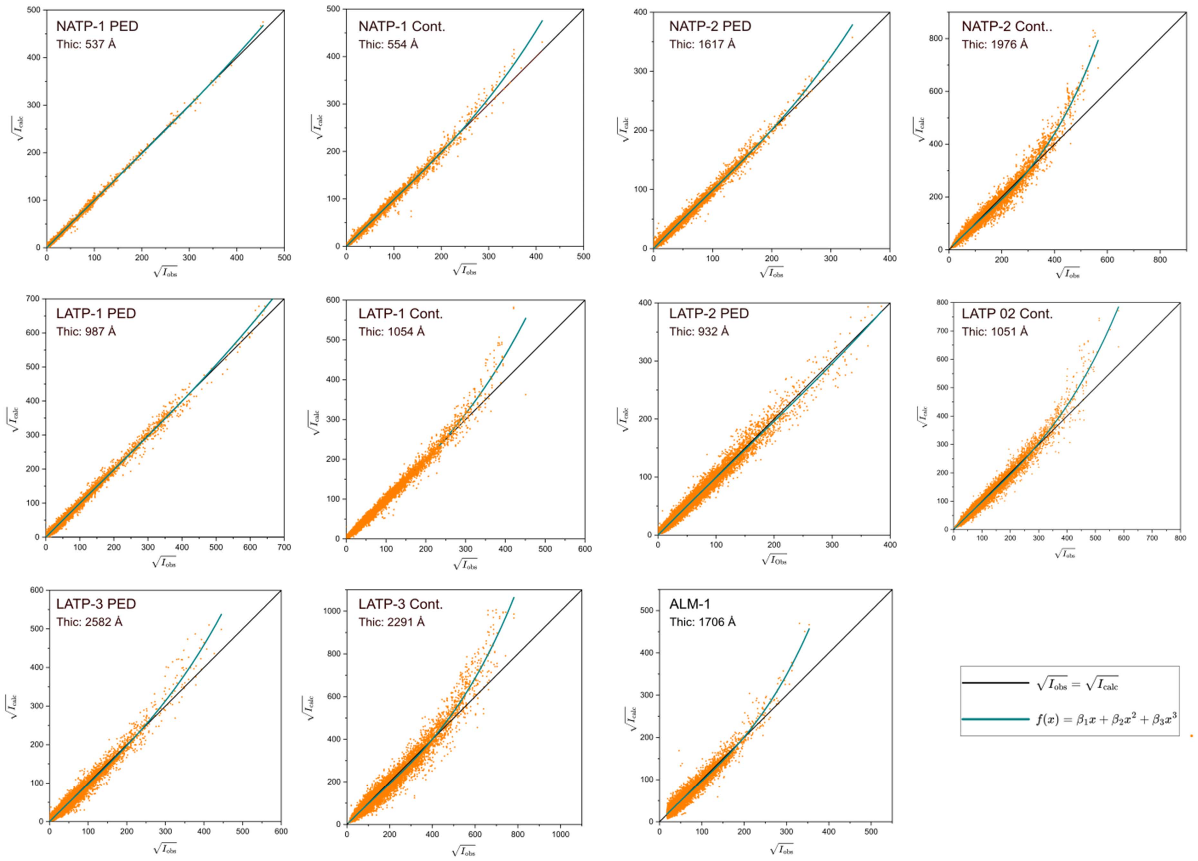

Figure S7 $I_{calc}{}^{1/2}$ against $I_{obs}{}^{1/2}$ for the crystals NATP-1, NATP-2, LATP-1, LATP-2, LATP-3 and ALM-1. A cubic polynomial (green) was fitted against the reflection data, where strong reflections appear systematically over-approximated and deviating from the ideal linear condition $I_{obs}{}^{1/2} = I_{calc}{}^{1/2}$ (black).

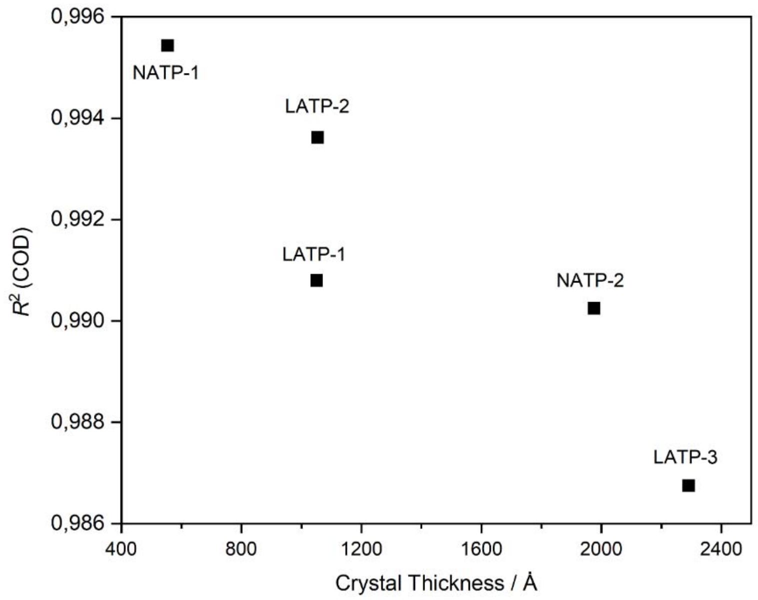

Figure S8 Variance of the cubic regression model plotted for different sample thicknesses.

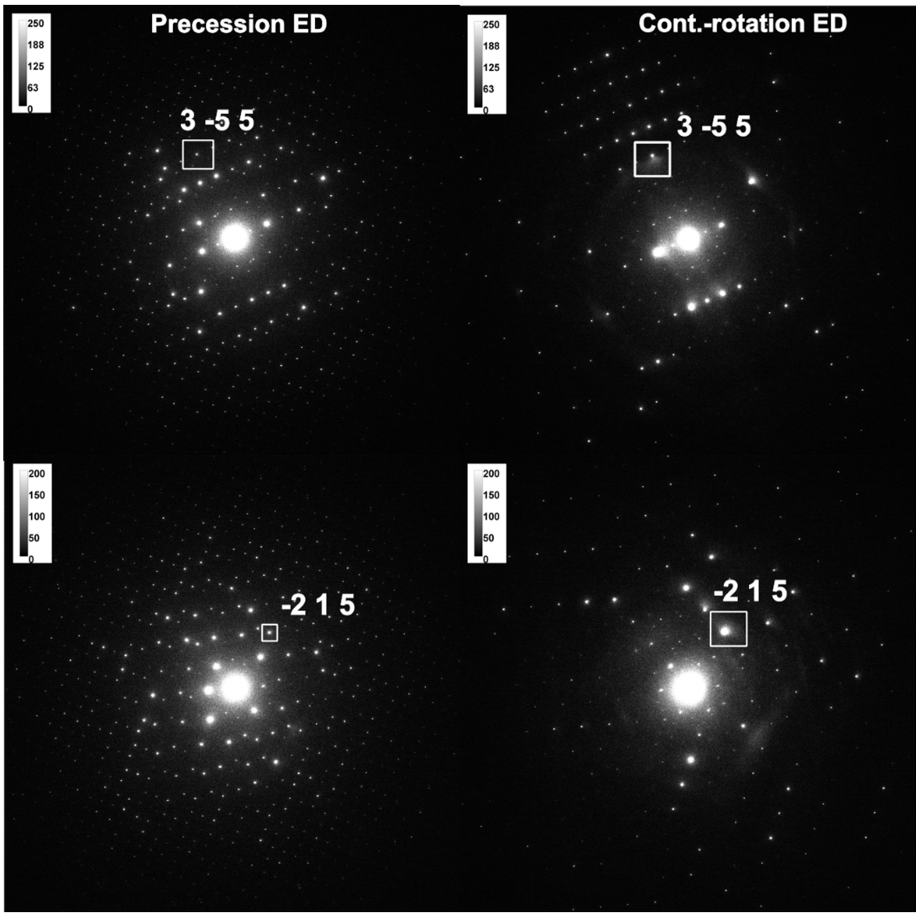

Figure S9 Raw diffraction patterns for reflections shown in Fig. 5 e and f. All frames are displayed at the same white value.
